# Supplementary material for: The archaeal class Nitrososphaeria is a key component of the reproductive microbiome in sponges during gametogenesis
Source: mBio. 2025 May 1;16(6):e02019-24. doi: 10.1128/mbio.02019-24 (PMC12153309; doi:10.1128/mbio.02019-24)
Supplement: Figure S1 — PCoA plots. [file mbio.02019-24-s0001.pdf]

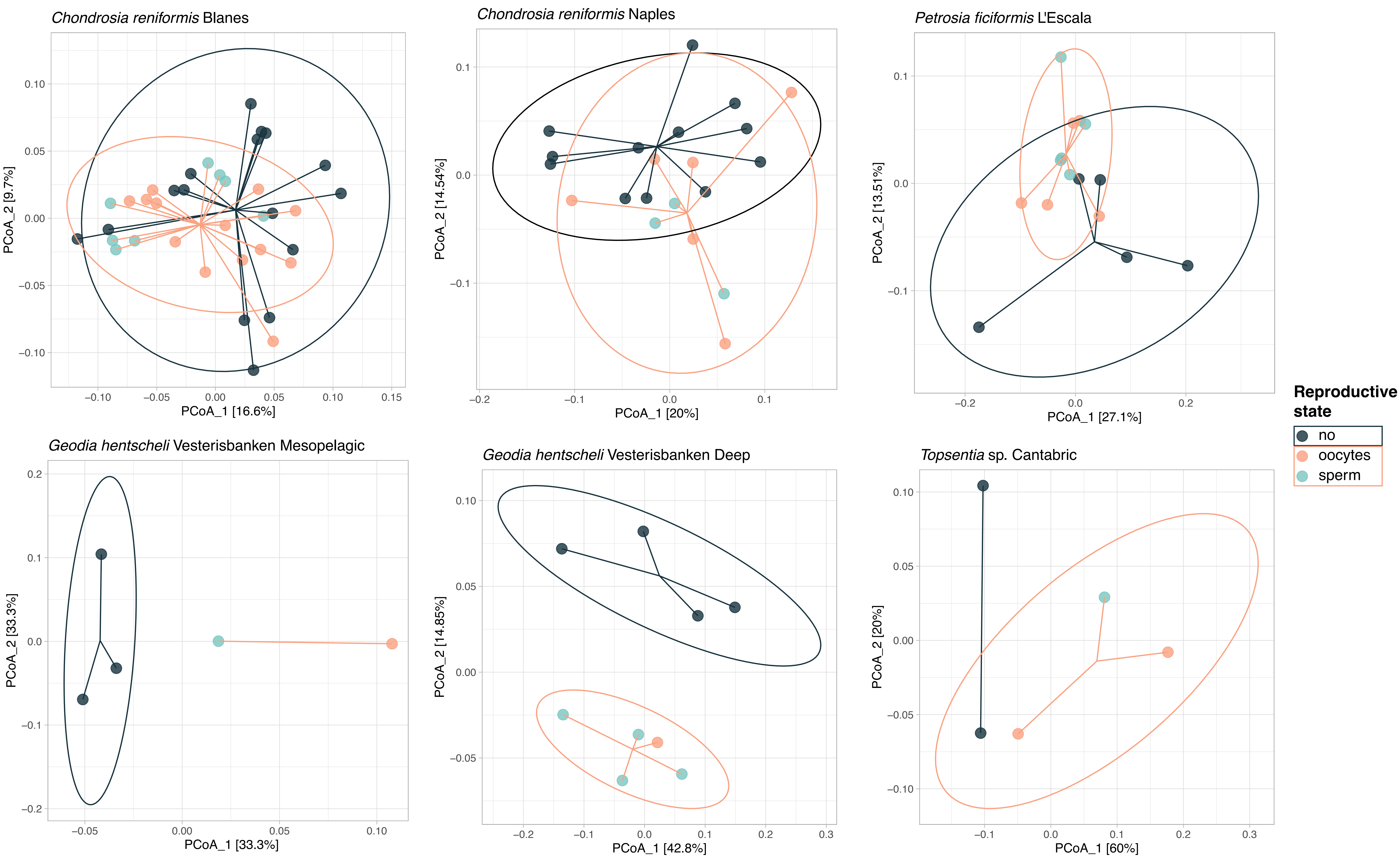

Figure S1. Principal coordinate analysis (PCoA) plots based on Bray-Curtis dissimilarity index of the microbial composition separated for each species and location, coloured according to their reproductive stage. Plots for G. macandrewii and P.ficiformis from Naples are shown in Figure 4 and 5 in the main text. Variation explained by the first two axes is indicated as %.
