# Supplementary material for: The archaeal class Nitrososphaeria is a key component of the reproductive microbiome in sponges during gametogenesis
Source: mBio. 2025 May 1;16(6):e02019-24. doi: 10.1128/mbio.02019-24 (PMC12153309; doi:10.1128/mbio.02019-24)
Supplement: Figure S2 — G. macandrewii ZOTUs. [file mbio.02019-24-s0002.pdf]

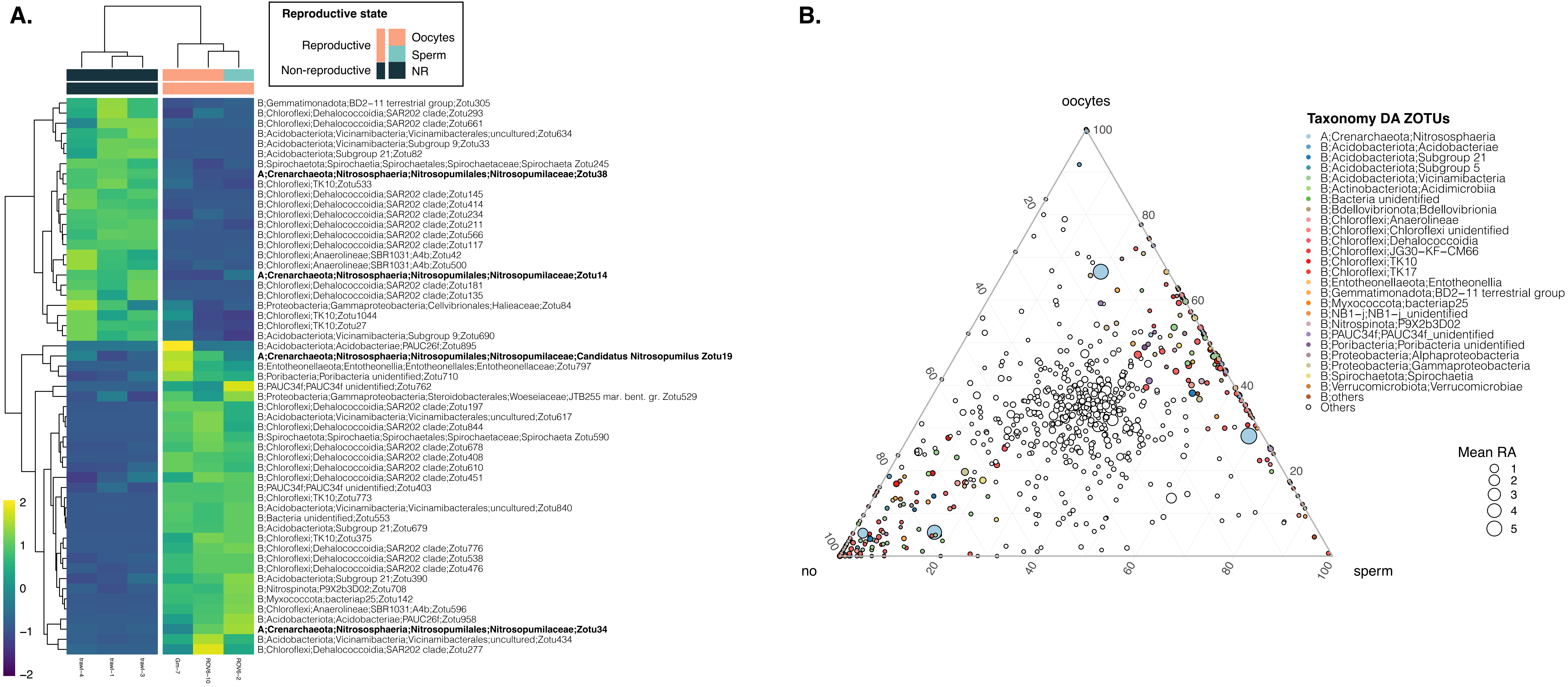

Figure S2: G. macandrewii. ZOTUs **A.** Heatmap of the most abundant differentially abundant (DA) ZOTUs between reproductive and non-reproductive individuals of G. macandrewii, with log transformed abundances represented in the colour temperature bar. Microbial ZOTUs are organized according to a hierarchical clustering based on Bray-Curtis Dissimilarity matrices. Sponge individuals (x-axis) are coloured according to Reproductive stage. Row names indicate the taxonomy of the DA ZOTUs (A: Archaea, B: Bacteria), in bold the 4 most abundant DA ZOTUs. **B.** Ternary plot of the ZOTUs distribution along reproductive stages (oocytes, sperm, non-reproductive) for G. macandrewii. Each circle represents a ZOTU and its size is proportional to its mean relative abundance across all samples. Only DA ZOTUs are coloured according to their taxonomy at class level.
