# Supplementary material for: The archaeal class Nitrososphaeria is a key component of the reproductive microbiome in sponges during gametogenesis
Source: mBio. 2025 May 1;16(6):e02019-24. doi: 10.1128/mbio.02019-24 (PMC12153309; doi:10.1128/mbio.02019-24)
Supplement: Supplemental text — Statistical analyses for beta and alpha diversity. [file mbio.02019-24-s0003.docx]

**Supplementary Text File:** Statistical analyses for beta and alpha (Shannon and Inverse Simpson) diversity. All the analyses are conducted first, for the whole dataset with the factor’s species and site, and then, for each species separately.

**All dataset (Betadiversity)**

***Permutest for species***

Permutation test for homogeneity of multivariate dispersions

Permutation: free

Number of permutations: 999

| Response: | Distances | |  |  |  |  |
| --- | --- | --- | --- | --- | --- | --- |
|  | Df | Sum Sq | Mean Sq | F | N.Perm | Pr(>F) |
| Groups | 4 | 0.085 | 0.021 | 8.87 | 999 | **0.001 ***** |
| Residuals | 101 | 0.243 | 0.002 |  |  |  |

---

Signif. codes: 0 ‘***’ 0.001 ‘**’ 0.01 ‘*’ 0.05 ‘.’ 0.1 ‘ ’ 1

***Permanova for species***

Permutation test for adonis under reduced model

Terms added sequentially (first to last)

Permutation: free

Number of permutations: 999

adonis2(formula = counts_bc ~ Species, data = Infotable)

|  | Df | SumOfSqs | R2 | F | Pr(>F) |
| --- | --- | --- | --- | --- | --- |
| Species | 4 | 28.5 | 0.89 | 212.18 | **0.001** *** |
| Residual | 101 | 3.39 | 0.11 |  |  |
| Total | 105 | 31.89 | 1 |  |  |

---

Signif. codes: 0 ‘***’ 0.001 ‘**’ 0.01 ‘*’ 0.05 ‘.’ 0.1 ‘ ’ 1

***Permanova for species and site (nested factors)***

Permutation test for adonis under reduced model

Terms added sequentially (first to last)

Permutation: free

Number of permutations: 999

adonis2(formula = counts_bc ~ Species * Site, data = Infotable)

|  | Df | SumOfSqs | R2 | F | Pr(>F) |
| --- | --- | --- | --- | --- | --- |
| Species | 4 | 28.5 | 0.89 | 258.84 | **0.001** *** |
| Species:Site | 2 | 0.66 | 0.02 | 12.11 | **0.001** *** |
| Residual | 99 | 2.72 | 0.08 |  |  |
| Total | 105 | 31.89 | 1 |  |  |

---

Signif. codes: 0 ‘***’ 0.001 ‘**’ 0.01 ‘*’ 0.05 ‘.’ 0.1 ‘ ’ 1

***P. ficiformis* (Site effect)**

***Permutest for Site***

Permutation test for homogeneity of multivariate dispersions

Permutation: free

Number of permutations: 999

| Response: | Distances | |  |  |  |  |
| --- | --- | --- | --- | --- | --- | --- |
|  | Df | Sum Sq | Mean Sq | F | N.Perm | Pr(>F) |
| Groups | 1 | 0.042 | 0.042 | 26.29 | 999 | **0.001 ***** |
| Residuals | 21 | 0.033 | 0.001 |  |  |  |

---

Signif. codes: 0 ‘***’ 0.001 ‘**’ 0.01 ‘*’ 0.05 ‘.’ 0.1 ‘ ’ 1

***Permanova for Site***

Permutation test for adonis under reduced model

Terms added sequentially (first to last)

Permutation: free

Number of permutations: 999

adonis2(formula = counts_bc ~ Site, data = Infotable)

|  | Df | SumOfSqs | R2 | F | Pr(>F) |
| --- | --- | --- | --- | --- | --- |
| Species | 1 | 0.43 | 0.33 | 10.5 | **0.001** *** |
| Residual | 21 | 0.86 | 0.66 |  |  |
| Total | 22 | 1.29 | 1 |  |  |

---

Signif. codes: 0 ‘***’ 0.001 ‘**’ 0.01 ‘*’ 0.05 ‘.’ 0.1 ‘ ’ 1

***C. reniformis* (Site effect)**

***Permutest for Site***

Permutation test for homogeneity of multivariate dispersions

Permutation: free

Number of permutations: 999

| Response: | Distances | |  |  |  |  |
| --- | --- | --- | --- | --- | --- | --- |
|  | Df | Sum Sq | Mean Sq | F | N.Perm | Pr(>F) |
| Groups | 1 | 0.004 | 0.004 | 11.158 | 999 | **0.005 ***** |
| Residuals | 56 | 0.022 | 0.0004 |  |  |  |

---

Signif. codes: 0 ‘***’ 0.001 ‘**’ 0.01 ‘*’ 0.05 ‘.’ 0.1 ‘ ’ 1

***Permanova for Site***

Permutation test for adonis under reduced model

Terms added sequentially (first to last)

Permutation: free

Number of permutations: 999

adonis2(formula = counts_bc ~ Site, data = Infotable)

|  | Df | SumOfSqs | R2 | F | Pr(>F) |
| --- | --- | --- | --- | --- | --- |
| Species | 1 | 0.23 | 0.15 | 10.38 | **0.001** *** |
| Residual | 56 | 1.26 | 0.84 |  |  |
| Total | 57 | 1.49 | 1 |  |  |

---

Signif. codes: 0 ‘***’ 0.001 ‘**’ 0.01 ‘*’ 0.05 ‘.’ 0.1 ‘ ’ 1

**All dataset (Alphadiversity)**

***Shannon: Permanova for species***

Permutation test for adonis under reduced model

Terms added sequentially (first to last)

Permutation: free

Number of permutations: 999

adonis2(formula = shannonH ~ Species, data = Infotable)

|  | Df | SumOfSqs | R2 | F | Pr(>F) |
| --- | --- | --- | --- | --- | --- |
| Species | 4 | 0.504 | 0.93 | 359.38 | **0.001** *** |
| Residual | 101 | 0.035 | 0.06 |  |  |
| Total | 105 | 0.54 | 1 |  |  |

---

Signif. codes: 0 ‘***’ 0.001 ‘**’ 0.01 ‘*’ 0.05 ‘.’ 0.1 ‘ ’ 1

***InvSimp: Permanova for species***

Permutation test for adonis under reduced model

Terms added sequentially (first to last)

Permutation: free

Number of permutations: 999

adonis2(formula = shannonH ~ Species, data = Infotable)

|  | Df | SumOfSqs | R2 | F | Pr(>F) |
| --- | --- | --- | --- | --- | --- |
| Species | 4 | 2.2 | 0.507 | 25.97 | **0.001** *** |
| Residual | 101 | 2.14 | 0.492 |  |  |
| Total | 105 | 4.35 | 1 |  |  |

---

Signif. codes: 0 ‘***’ 0.001 ‘**’ 0.01 ‘*’ 0.05 ‘.’ 0.1 ‘ ’ 1

**Species and locations (Betadiversity)**

Summary table showing the results of Permutest and Permanova for each species and location. Permanova was first performed comparing reproductive vs non-reproductive individuals, and then comparing different reproductive stages (oocytes, sperm and non-reproductive).

| **Species** | **Site** | **n** | **Permutest** | | **Permanova (Repro vs NR)** | | **Permanova**  **(Repro Stage)** | |
| --- | --- | --- | --- | --- | --- | --- | --- | --- |
| *Geodia macandrewii* | North Atlantic | 6 | F | 0.53 | R^2^ | 0.70 | R^2^ | 0.79 |
|  |  |  | p-val | 0.501 | p-val | 0.1 | p-val | **0.05*** |
| *Petrosia ficiformis* | Naples | 8 | F | 0.14 | R^2^ | 0.29 | R^2^ | 0.29 |
|  |  |  | p-val | 0.746 | p-val | 0.029 | p-val | **0.03*** |
|  | L’Escala | 15 | F | 1.99 | R^2^ | 0.11 | R^2^ | 0.18 |
|  |  |  | p-val | 0.193 | p-val | **0.032**** | p-val | 0.07 |
| *Chondrosia reniformis* | Blanes | 37 | F | 2.81 | R^2^ | 0.03 | R^2^ | 0.06 |
|  |  |  | p-val | 0.103 | p-val | 0.18 | p-val | 0.12 |
|  | Naples | 21 | F | 2.65 | R^2^ | 0.07 | R^2^ | 0.1 |
|  |  |  | p-val | 0.139 | p-val | 0.08 | p-val | 0.43 |
| *Geodia hentscheli* | Vesterisbanken (Deep) | 9 | F | 0.74 | R^2^ | 0.15 | R^2^ | 0.24 |
|  |  |  | p-val | 0.43 | p-val | 0.19 | p-val | 0.5 |
|  | Vesterisbanken (Mesopelagic) | 5 | F | 0.11 | R^2^ | 0.29 | R^2^ | 0.54 |
|  |  |  | p-val | 0.608 | p-val | 0.1 | p-val | 0.2 |
| *Topsentia sp.* | Cantabric | 5 | F | 0.4 | R^2^ | 0.41 | R^2^ | 0.51 |
|  |  |  | p-val | 0.608 | p-val | 0.2 | p-val | 0.46 |

---

Signif. codes: 0 ‘***’ 0.001 ‘**’ 0.01 ‘*’ 0.05 ‘.’ 0.1 ‘ ’ 1

**Species and locations (Alphadiversity)**

| **Species** | **Site** | **Index** | **Normality** (p-val) | **Test** | **p-value** | **F-value** |
| --- | --- | --- | --- | --- | --- | --- |
| *Geodia macandrewii* | North Atlantic | Shannon | 0.03113 | Krukal-Wallis | 0.8273 |  |
|  |  | Richness | 0.2068 | Anova | 0.173 | 2.738 |
| *Petrosia ficiformis* | Naples | Shannon | 0.7816 | Anova | 0.667 | 0.205 |
|  |  | Richness | 0.3699 | Anova | 0.175 | 2.365 |
|  | L’Escala | Shannon | 0.4984 | Anova | 0.0718 | 3.841 |
|  |  | Richness | 0.1186 | Anova | 0.287 | 1.231 |
| *Chondrosia reniformis* | Blanes | Shannon | 0.0328 | Anova | **0.00948 **** | 7.536 |
|  |  | Richness | 0.4252 | Anova | **0.0147 *** | 6.583 |
|  | Naples | Shannon | 0.2316 | Anova | 0.869 | 0.028 |
|  |  | Richness | 0.3551 | Anova | 0.766 | 0.091 |
| *Geodia hentscheli* | Vesterisbanken (deep) | Shannon | 0.06497 | Anova | 0.231 | 1.722 |
|  |  | Richness | 0.7189 | Anova | 0.548 | 0.398 |
|  | Vesterisbanken (shallow) | Shannon | 0.23 | Anova | 0.584 | 0.375 |
|  |  | Richness | 0.2038 | Anova | **0.0158 *** | 24.5 |
| *Topsentia sp.* | Cantabric | Shannon | 0.8408 | Anova | 0.101 | 5.489 |
|  |  | Richness | 0.5814 | Anova | 0.0935 | 5.894 |

---

Signif. codes: 0 ‘***’ 0.001 ‘**’ 0.01 ‘*’ 0.05 ‘.’ 0.1 ‘ ’ 1

**Species and locations (Distance to centroid)**

| **Species** | **Site** | **Normality** (p-val) | **Test** | **p-value** | **F-value** |
| --- | --- | --- | --- | --- | --- |
| *Geodia macandrewii* | North Atlantic | 0.253 | Anova | 0.505 | 0.534 |
| *Petrosia ficiformis* | Naples | 0.7119 | Anova | 0.723 | 0.138 |
|  | L’Escala | 0.0891 | Anova | 0.181 | 1.995 |
| *Chondrosia reniformis* | Blanes | 0.4995 | Anova | 0.103 | 2.805 |
|  | Naples | 0.914 | Anova | 0.12 | 2.656 |
| *Geodia hentscheli* | Vesterisbanken (deep) | 0.06798 | Anova | 0.417 | 0.744 |
|  | Vesterisbanken (shallow) | 0.3091 | Anova | 0.756 | 0.116 |
| *Topsentia sp.* | Cantabric | 0.7301 | Anova | 0.572 | 0.4 |

---

Signif. codes: 0 ‘***’ 0.001 ‘**’ 0.01 ‘*’ 0.05 ‘.’ 0.1 ‘ ’ 1
