# Supplementary material for: The archaeal class Nitrososphaeria is a key component of the reproductive microbiome in sponges during gametogenesis
Source: mBio. 2025 May 1;16(6):e02019-24. doi: 10.1128/mbio.02019-24 (PMC12153309; doi:10.1128/mbio.02019-24)
Supplement: Figure S3 — Treemap of the upregulated functions in reproductive individuals. [file mbio.02019-24-s0004.pdf]

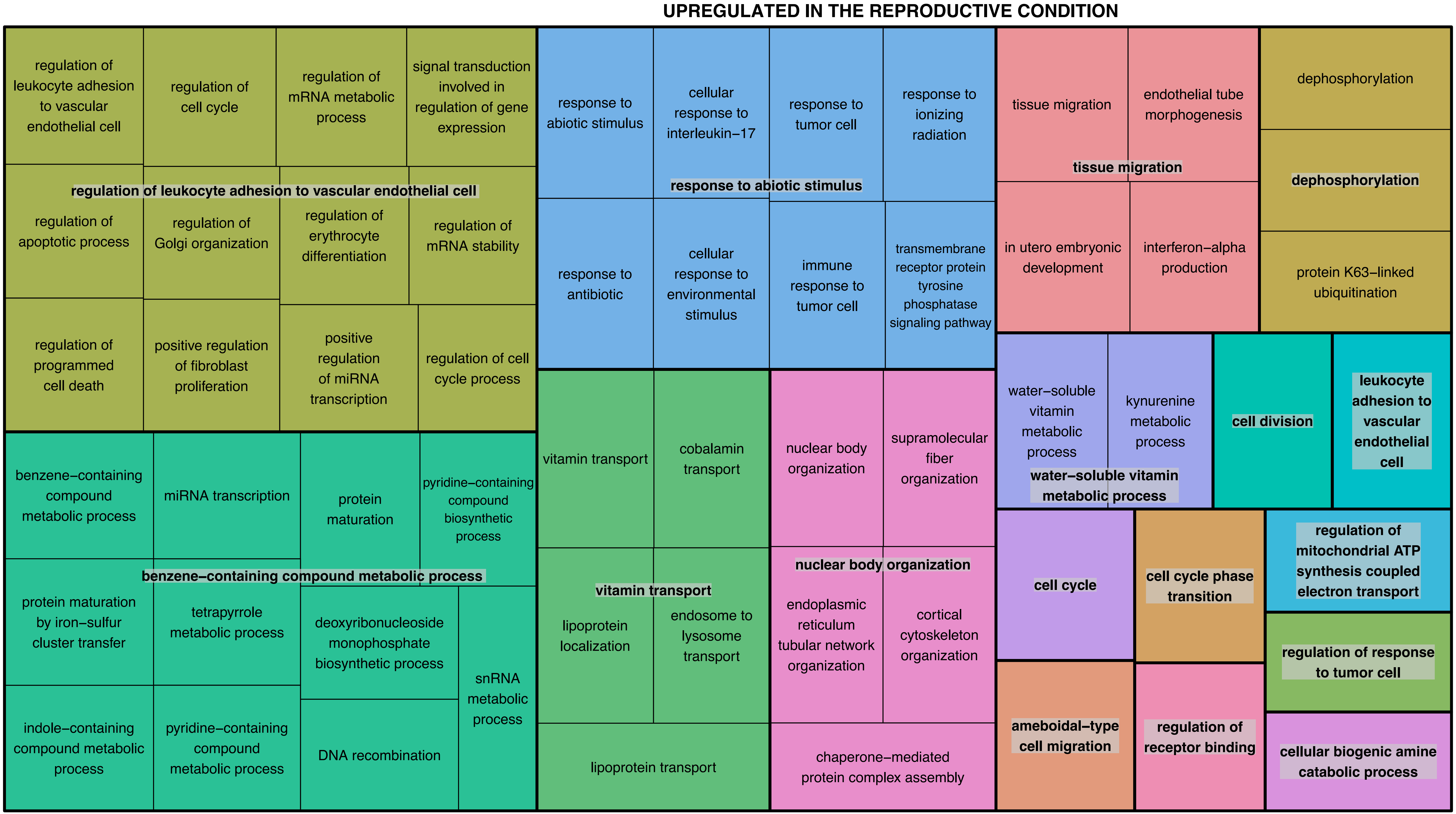

Figure S3. Treemap of the upregulated functions in reproductive individuals. Size of the squares is proportional to the TMM expression values of each function.
